# Malignant complications of celiac disease: a case series and review of the literature

**DOI:** 10.1186/s13256-022-03682-3

**Published:** 2022-12-12

**Authors:** Barbora Packova, Pavel Kohout, Milan Dastych, Jitka Prokesova, Tomas Grolich, Radek Kroupa

**Affiliations:** 1 Department of Internal Medicine and Gastroenterology, University Hospital Brno, Faculty of Medicine, Masaryk University, Jihlavska 20, 62500 Brno, Czech Republic; 2 Department of Internal Medicine, Third Faculty of Medicine, Charles University Prague and Teaching Thomayer Hospital, 14059 Prague, Czech Republic; 3 Department of Surgery, University Hospital Brno, Faculty of Medicine, Masaryk University, 62500 Brno, Czech Republic

**Keywords:** Celiac disease, Malignancy, Complication, Lymphoma, Carcinoma, Case report

## Abstract

**Background:**

Celiac disease is an immune-mediated enteropathy triggered by gluten in genetically susceptible individuals. Diagnosis is based on evaluating specific autoantibodies and histopathologic findings of duodenal biopsy specimens. The only therapy for celiac disease is a gluten-free diet. Celiac disease can be complicated by malnutrition, other autoimmune diseases, refractoriness to treatment, and gastrointestinal tumors. This article presents seven cases of malignancies in patients with celiac disease. Its objective is to raise awareness of the malignant complications of celiac disease, leading to earlier diagnosis and improved outcomes.

**Case presentation:**

Seven cases of malignant complications of celiac disease occurred among 190 patients followed at the Department of Internal Medicine and Gastroenterology, University Hospital Brno from 2014 to 2021. We describe these cases and the presentation, diagnostic process, course, management, and outcomes for each, along with proposed potential risk factors of malignant complications. There was one Caucasian man who was 70 years old and six Caucasian women who were 36, 46, 48, 55, 73, and 82 years old in our cohort. Of the seven cases of malignancies in our cohort, four patients were diagnosed with small bowel adenocarcinoma, one with diffuse large B-cell lymphoma, one with carcinoma of the tongue, and one with colorectal carcinoma.

**Conclusions:**

Malignancies occurred in 3.7% of patients followed up for celiac disease. Awareness of the malignant complications of celiac disease, risk factors, presentation, and disease course could lead to earlier diagnosis and improved outcomes.

## Background

Celiac disease (CD) is an immune-mediated enteropathy triggered by gluten in genetically susceptible individuals. The diagnosis is based on specific autoantibodies—anti-tissue transglutaminase (a-TTG), anti-deaminated gliadin peptides (a-DGP), and endomysial antibodies—and a duodenal biopsy specimen showing elevated levels of intraepithelial lymphocytes (Marsh 1 according to the Marsh–Oberhuber classification), hyperplasia of crypts (Marsh 2), and partial, moderate, or complete atrophy of villi (Marsh 3a, b, or c). The only therapy for CD is a gluten-free diet (GFD). CD can be complicated by malnutrition, other autoimmune diseases, refractoriness to treatment, and gastrointestinal tumors. Enteropathy-associated T-cell lymphoma (EATL) has the highest association with CD [relative risk (RR) 35.8], followed by adenocarcinoma of the small intestine (RR 14.4) and duodenum (RR 10.2) [1]. CD is also associated with an increased risk of other types of non-Hodgkin lymphoma [2–6], and in some studies, esophageal [5, 7–10] and oropharyngeal [7] carcinoma, hepatobiliary and pancreatic carcinoma [7, 8], and, arguably, colorectal carcinoma (CRCA) [7].

This article presents seven cases of malignancies in patients with CD, including the presentation, diagnostic process, course, management, outcomes, and proposed potential risk factors for each. Awareness of these malignant complications is insufficient. This article is unique in the number and variety of malignant complications of CD presented. Its objective is to raise awareness of the malignant complications of CD, leading to earlier diagnosis and improved outcomes.

## Case presentations

A total of 190 patients with CD were followed at the Department of Internal Medicine and Gastroenterology, University Hospital Brno, from 2014 to 2021. All patients followed for a CD diagnosis were included in the general study population, and none were excluded. In this group, 163 (85.8%) patients were women, and the mean (± SD) age at diagnosis was 32.4 ± 15.9 years. Seven cases of malignant complications of CD were detected in the group during this period. For each case, we present the nature of presentation, diagnostic process, course of disease, therapy, and outcome. All the patients signed informed consent regarding anonymous data collection, and the study protocol was approved by the Multicentric Ethical Committee University Hospital Brno, No. 03-180919/EK.

### Case 1

The first case was a 36-year-old Caucasian woman diagnosed at 6 years of age with CD, which initially presented with diarrhea and failure to thrive. There were two cases of CD but no case of gastrointestinal malignity in her family history. Information was unavailable on the CD stage at the time of diagnosis. She started a GFD, and disease was stable for decades. At age 35 years, the patient presented with abdominal pain and nausea. Gastroscopy showed stenosis of the duodenal bulb with villous atrophy (Marsh 3a) on biopsy specimen. The patient was diagnosed with refractory CD type I and started on corticotherapy. The stenosis progressed despite the therapy, and a follow-up biopsy specimen obtained after 3 months revealed adenocarcinoma of the duodenum. There were no signs of metastases on computed tomography (CT). Hemipancreatoduodenectomy was performed, with R0 resection of grade 3 adenocarcinoma pT4N1M0 (stage III). The patient received adjuvant chemotherapy; however, over the next 5 years, she required several courses of stereotactic radiotherapy and chemotherapy for disease progression and metastasis. However, she has been cancer-free for 36 months since last recurrence.

### Case 2

The second case was an 82-year-old Caucasian woman investigated for abdominal pain. Stenosis of the duodenum was found on gastroscopy, and a biopsy specimen revealed adenocarcinoma within the terrain of CD Marsh 3c. CT showed a tumor of the D2/3 duodenum with infiltration of adjacent fat, lymph nodes, and possibly the inferior vena cava without apparent metastases (stage III). The interdisciplinary team decided on palliative gastroenteroanastomosis due to the patient’s age and comorbidities; she underwent surgery without complications. The patient received analgesic and nutritional treatment in a palliative regime and died 9 months after the diagnosis. She had a history of sideropenic anemia, but no history of abdominal symptoms or weight loss before adenocarcinoma was diagnosed. However, her daughter, two granddaughters, and two great-granddaughters had CD.

### Case 3

**Fig. 1:**
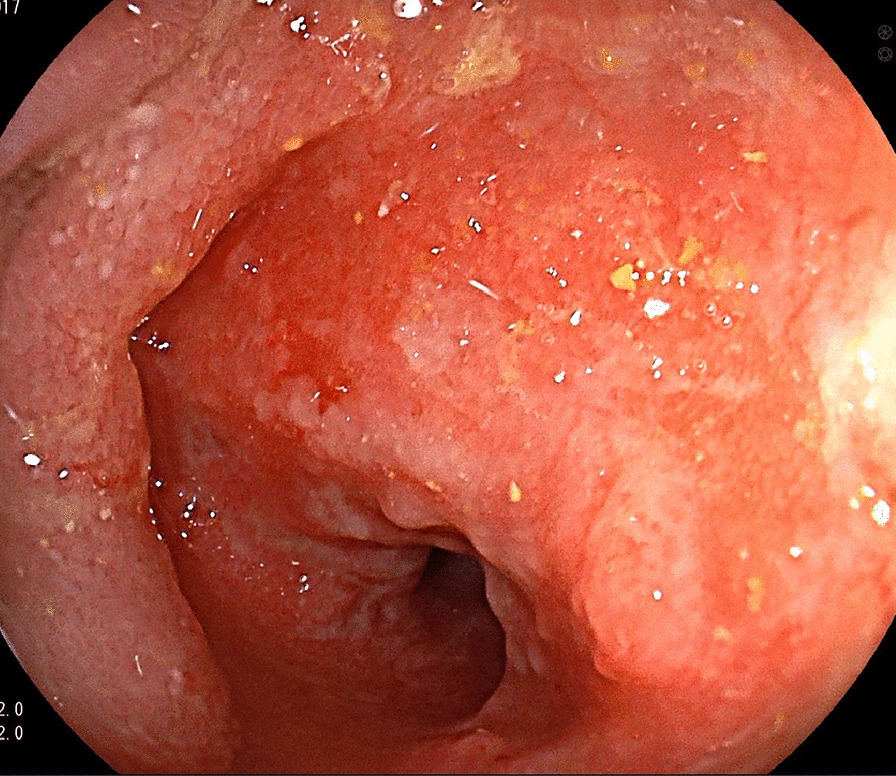
Gastroscopic view of adenocarcinoma of proximal jejunum. Provided by Radek Kroupa M.D. from archive of Department of Internal Medicine and Gastroenterology, University Hospital Brno

**Fig. 2:**
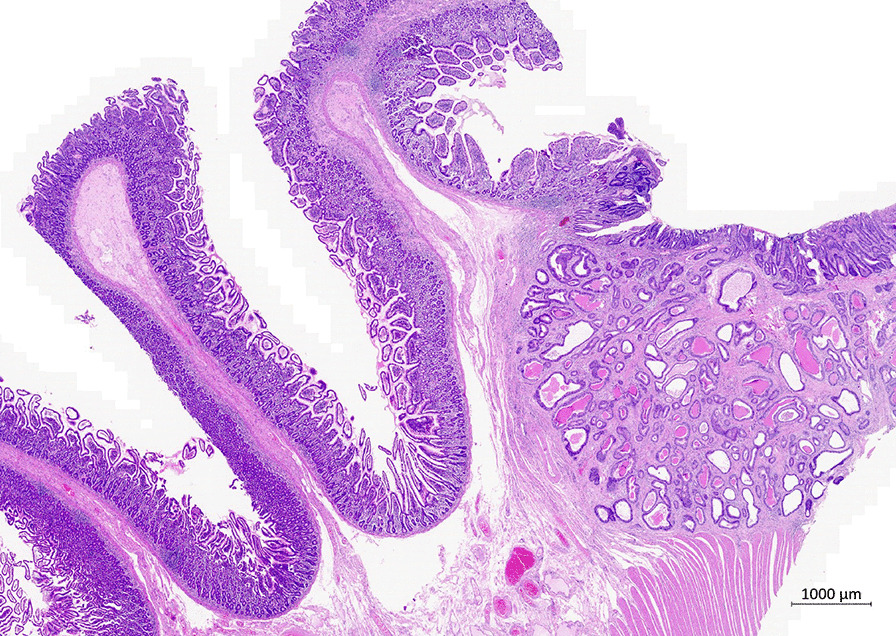
Adenocarcinoma of the oral jejunum, intestinal type (right). Intestinal villi without atrophy, but with increased number of intraepithelial lymphocytes (left). Hematoxylin + eosin, 200×. Provided by Leos Kren, Ass. Prof., M.D., Ph.D. from archive of Department of Pathology, University Hospital Brno

The third patient was a 70-year-old Caucasian man diagnosed with CD at the age of 64 years. He was investigated for sideropenic anemia, with only meteorism as an abdominal symptom, and his daughter had CD. Mesenterial lymphadenopathy was found on abdominal ultrasonography, with positive a-TTG and Marsh 3a on a biopsy specimen from the distal duodenum. Positron emission tomography/computed tomography (PET/CT) was performed because of mesenterial lymphadenopathy, with no abnormal findings. The patient started a GFD and remained compliant and clinically stable. After 6 years, the patient started to experience abdominal pain, vomiting, and the reappearance of sideropenic anemia. Gastroscopy detected stenosis of the oral jejunum (Fig. 1), and histological analysis of a biopsy specimen revealed adenocarcinoma. Unaffected small bowel tissue did not show signs of active CD (Marsh 1). The stenosis was radically resected with a final diagnosis of acinar adenocarcinoma grade 2, pT3N0M0 (stage II) (Fig. 2), and the patient received adjuvant chemotherapy with capecitabine, which was stopped after the first series due to diarrhea and abdominal pain. The patient was simultaneously diagnosed with acinar adenocarcinoma of the prostate, T2NxMx, which was treated with radiotherapy and hormonal therapy. The patient has been in remission for 50 months.

### Case 4

**Fig. 3:**
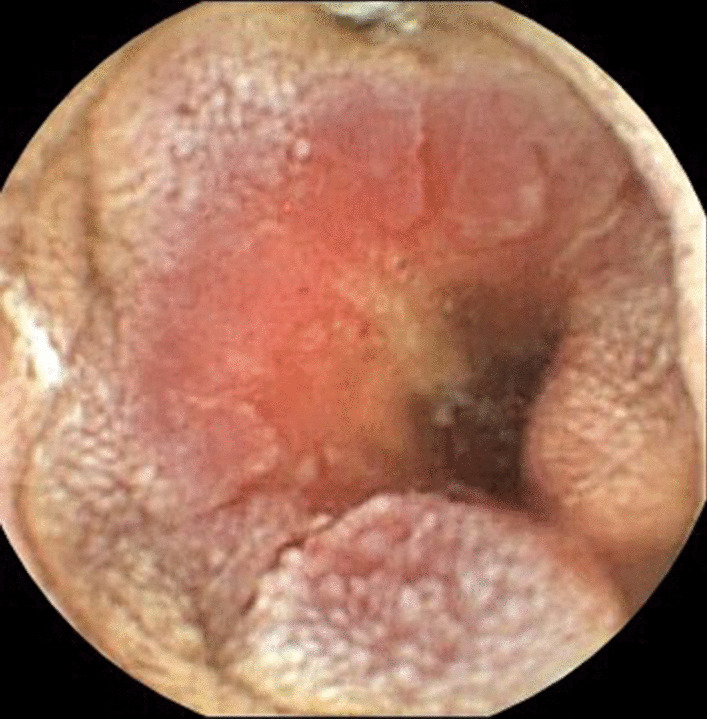
Small bowel adenocarcinoma in view by video capsule enteroscopy. Provided by Milan Dastych M. D. from archive of Department of Internal Medicine and Gastroenterology, University Hospital Brno

**Fig. 4:**
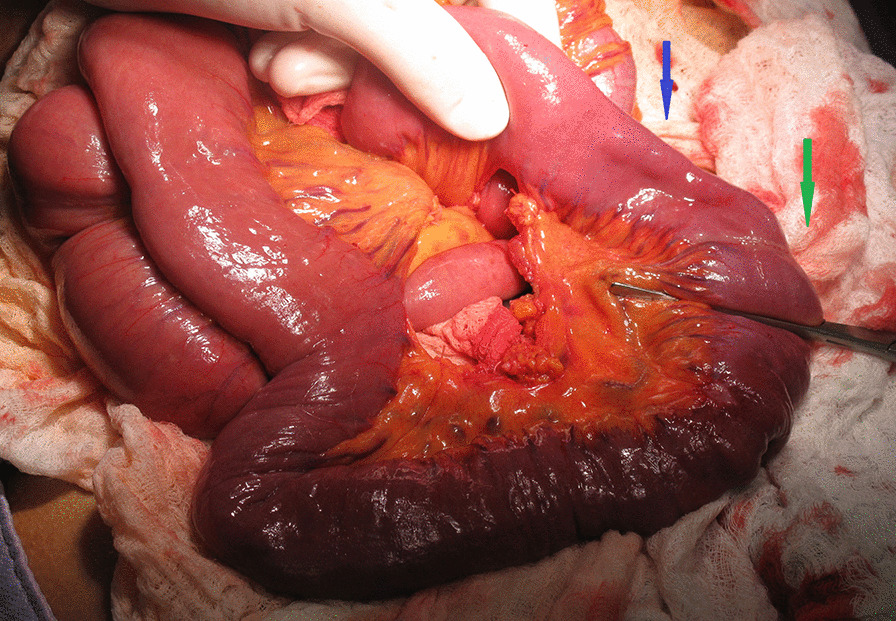
Perioperative view of infiltration of jejunum (green arrow) with the lodged capsule (blue arrow). Provided by Tomas Grolich M. D., Department of Surgery, University Hospital Brno

The fourth case is a 55-year-old Caucasian woman diagnosed with CD at the age of 53 years. She had Sjögren’s syndrome and a family history of CD with no cases of malignity in her family history. She had positive a-TTG and a-DGP, with Marsh 3b on duodenal biopsy specimen at the time of diagnosis. She started a GFD, and after 2 years of clinical stability, she presented with sideropenic anemia. Gastroscopy and colonoscopy images were unremarkable, while a duodenal biopsy specimen showed stable Marsh 1 disease. Video capsule enteroscopy revealed exulcerated, stenotized infiltration of the jejunum, with signs of bleeding (Fig. 3). She underwent R0 resection of adenocarcinoma of the jejunum grade 2, pT3N1M0 (stage III) (Fig. 4). The patient received adjuvant chemotherapy with XELOX (capecitabine plus oxaliplatine). Disease progression was detected after 1 year, and she died 22 months after jejunal adenocarcinoma was diagnosed.

### Case 5

**Fig. 5:**
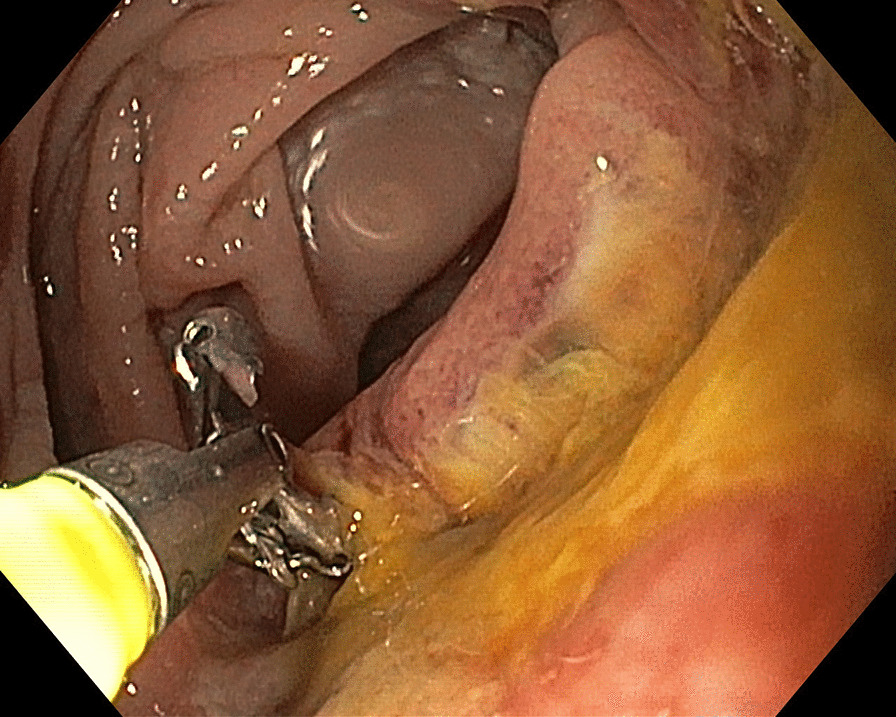
Gastroscopic view of diffuse large B-cell lymphoma infiltrating duodenal apex. Provided by Milan Dastych M. D. from archive of Department of Internal Medicine and Gastroenterology, University Hospital Brno

**Fig. 6:**
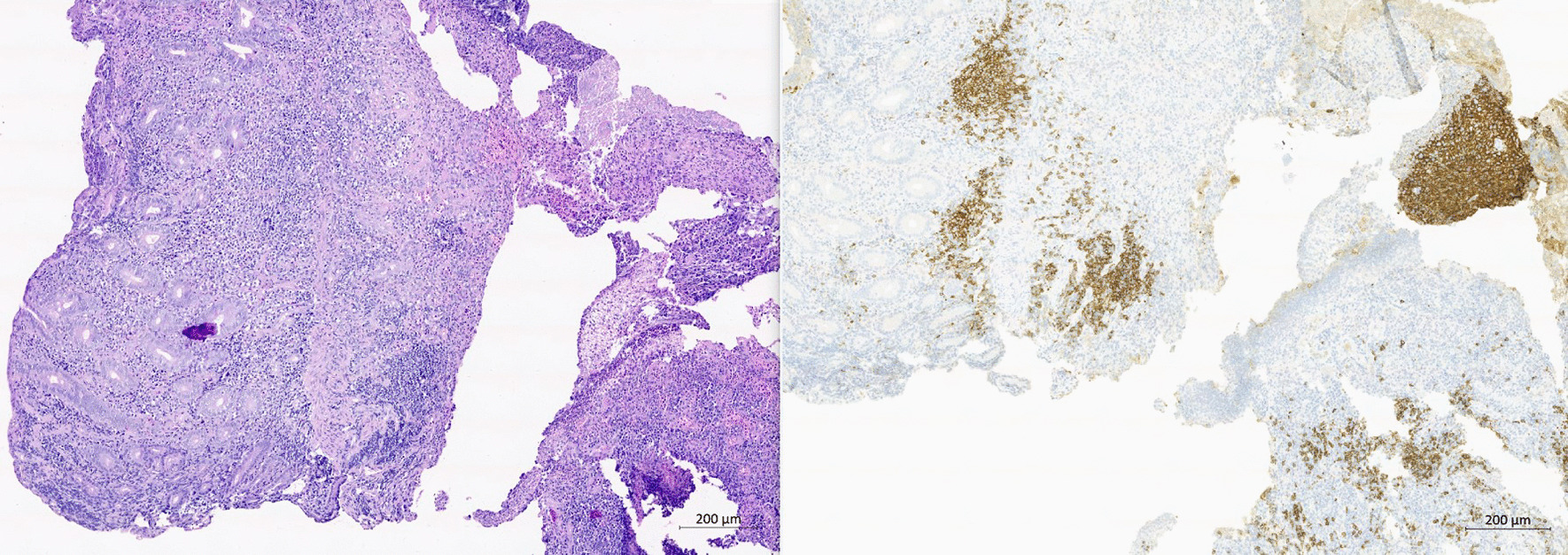
Biopsy of the duodenum, villi are flattened with intraepithelial lymphocytes. Regionally there are aggregates of large centrocytes in the lamina propria: infiltration by diffuse large B-cell lymphoma; hematoxylin + eosin, 200× (left). The large centrocytes stain with anti-CD 20 antibody; immunohistochemistry, 200× (right). Provided by Leos Kren, Ass. Prof., M.D., Ph.D. from archive of Department of Pathology, University Hospital Brno

The fifth case was a 73-year-old Caucasian woman who was investigated for diarrhea and malnutrition for many years. Idiopathic bowel disease, CD, and autoimmune pancreatitis were suspected but not confirmed. PET/CT showed mesenterial lymphadenopathy; however, other findings were negative, including a duodenal biopsy specimen and a fine needle aspiration specimen from the pancreas. There was a progression of malnutrition with the need for enteral nutrition when the patient was 71 years of age. Repeat evaluation showed mesenterial lymphadenopathy without progression on PET/CT, a new elevation of a-TTG, and villous atrophy on a duodenal biopsy specimen; the condition was determined to be florid CD. The patient’s condition improved, and she was stable on enteral nutrition for 16 months. However, the condition deteriorated with intestinal failure and a need for parenteral nutrition. There was an infiltration of the duodenum on follow-up PET/CT. Gastroscopy confirmed infiltration of the duodenum suggesting lymphoma (Fig. 5), and a biopsy specimen revealed diffuse large B-cell lymphoma (DLBCL) (Fig. 6), clinical stage IVB. The patient was treated with solumedrol and rituximab and then 6 cycles of  rituximab–cyclophosphamide–doxorubicine–vincristine–prednisolone (R-CHOP). Simultaneously, the patient was diagnosed with invasive lobular breast carcinoma, which was treated with hormonal therapy. The patient has been in remission for 45 months.

### Case 6

The sixth case was a 48-year-old Caucasian woman who experienced diarrhea and weight loss at 31 years of age. She was diagnosed with CD by positive autoantibodies and duodenal biopsy specimen, with no further details. She also had a diagnosis of gastroesophageal reflux disease and chronic pancreatitis, with a history of breast cancer in her mother at 45 years of age. However, she required a GFD in childhood, which was stopped during adolescence. The patient was not completely compliant with follow-up but claimed to be compliant with GFD. Her level of autoantibodies was negative, and a follow-up biopsy specimen from the distal duodenum showed Marsh 0. Odynophagia and sore throat progressed at age 47 years; however, she was not compliant with further investigation. There was transitory stabilization of the patient’s complaints; however, her odynophagia progressed, and paresis of the left twelfth cranial nerve appeared the following year. She was examined at the otorhinolaryngology and stomatology department, and infiltration of the distal tongue was diagnosed. The patient underwent resection of adenoid cystic carcinoma of the tongue body and radix, pT4apN0M0 (stage IV). However, radical resection was not feasible; she underwent a total of five operations and adjuvant radiotherapy. The patient is in remission 20 months after the end of radiotherapy.

### Case 7

The last case was a 46-year-old Caucasian woman diagnosed with CD at 39 years of age. The patient was investigated for CD due to difficulties treating type I diabetes mellitus. The initial examination revealed positive a-TTG, a-DGP, and Marsh 3b. The patient started a GFD, which promptly stabilized her diabetes. A control biopsy specimen of the duodenum obtained after 4 years due to remaining slight positivity of a-DGP produced a negative finding of Marsh 0; the level of autoantibodies completely normalized 5 years after the diagnosis. The patient was stable until 2 years later, when she developed hypogastric abdominal pain that was relieved after defecation. Colonoscopy showed stenosis of the colon sigmoideum, with adenocarcinoma on bioptic examination. CT showed infiltration of the colon sigmoideum with lymphadenopathy, and no other intraabdominal dissemination. Right-sided hemicolectomy with transversorectoanastomosis was performed, with a final diagnosis of adenocarcinoma of the colon descendens grade 1 with angioinvasion, pT3N1(2/28) M0 (stage III). The patient received adjuvant chemotherapy with oxaliplatine + capecitabine. Remission of disease was observed on surveillance PET/magnetic resonance (MR) performed 6 months after the operation. The patient had no risk factors for colorectal cancer, as only type II diabetes mellitus is considered a risk factor for CRCA. There were no other cases of CRCA in her family history.

## Discussion and conclusions

The overall risk of malignancy is not increased in CD [standardized incidence ratio (SIR) 0.97], but there is an increased risk of gastrointestinal malignancies (SIR 1.42) according to a recent study [11]. Malignant complications of CD are rare but have a very poor prognosis. One of the reasons for the unfavorable prognosis is the late diagnosis. Determining the patients at risk and studying the course of these complications could improve their outcomes. Malignant complications occurred in 3.7% (seven) of our patients (summarized in Table 1).

**Table 1:** Summary of the malignancies in celiac disease

Patient	Age at diagnosis of CD, years	Age at diagnosis of malignity, years	Persistent villous atrophy	Anemia	Type of malignity	Stage	Therapy	Outcome
1. Caucasian woman	6	36	Yes	No	SBA	III	Resection, chemotherapy, radiotherapy	Cancer free at 36 months
2. Caucasian woman	82	82	–	Yes	SBA	III	Palliation	Death in 9 months
3. Caucasian man	64	70	No	Yes	SBA	II	Resection, chemotherapy	Cancer free at 50 months
4. Caucasian woman	53	55	No	Yes	SBA	III	Resection, chemotherapy	Death in 22 months
5. Caucasian woman	71	73	Yes	Yes	DLBCL	IVB	Chemotherapy	Cancer free at 45 months
6. Caucasian woman	31	48	No	No	Carcinoma of tongue	IV	Resection, radiotherapy	Cancer free at 20 months
7. Caucasian woman	39	46	No	No	CRCA	III	Resection, chemotherapy	Cancer free at 6 months

*SBA* small bowel adenocarcinoma, *DLBCE* diffuse large blastic cell lymphoma, *CRCA* colorectal carcinoma

Information on the frequency of malignant complications of CD is sparse. Most studies evaluated the development of malignancy during the follow-up of patients with CD, ranging from 3–11% (0–7% in the case of lymphoma) [12–14]. The highest RR of malignant complication is in EATL, followed by small bowel adenocarcinoma (SBA) [1]*.* In our patients, the most frequent complication was SBA (4/7 cases); EATL was not detected in our patients. Although EATL has the highest association with CD, it is a very rare malignancy. Therefore, other malignancies, such as B-cell lymphoma, are more frequent than EATL. However, the incidence of SBA is not much higher than that of EATL; there were four cases in our cohort. The small number of patients probably caused this discrepancy. The same limitation prevented us from evaluating the risk factors of malignant complications. Male sex, the classic form of CD, older age, delay in diagnosis or untreated CD, HLA-DQ2 homozygosity, and persistent villous atrophy are risk factors for malignant complications [15–20]. Very recently, a large Swedish study concluded that the overall risk of malignancy was highest in those diagnosed with CD after age 60 years [21].

This article presented seven cases of cancer in patients with CD. In two cases (carcinoma of the tongue and CRCA), we can debate whether this was connected to CD. In the remaining five cases, there were three cases of persistent villous atrophy and two cases of classic CD, with only one case in a male patient; however, there were potentially four cases of delayed diagnosis or untreated CD, although this is difficult to evaluate. These four patients were 53, 64, 71, and 82 years of age at the time of CD diagnosis; one had been investigated for many years, while the other three had a family history of CD and atypical symptoms, which are known to delay final diagnosis. Moreover, four patients in our series had anemia during diagnosis of the malignant complication. We did not find any study evaluating this risk factor. However, anemia is a common presentation of gastrointestinal tumors.

Delayed diagnosis, untreated CD, and persistent villous atrophy are the most frequently mentioned risk factors of malignant complications [5, 7, 14, 22–25]. However, there are no recommendations for specific follow-up in these patients. Current guidelines recommend follow-up biopsy in symptomatic patients and possibly in patients aged over 40 years or those with initially severe presentations [26]. However, there is a trend in the search for non-invasive methods of follow-up. Our previous study suggested a combination of serology and experienced bowel ultrasound examination as a non-invasive method to predict persistent villous atrophy [27]. Close follow-up in cases of late-diagnosed or untreated CD or persistent villous atrophy, and prompt differential diagnosis of anemia in CD, could lead to earlier diagnosis and improved prognosis of malignant complications.

SBA is a rare disease with an incidence of 5.7/1,000,000 persons/year [28]. The tumor is usually diagnosed in the seventh decade of life [29]. Our patients were 36, 55, 70, and 82 years of age. An association with CD was detected in 13% of SBA cases [30]. High cellular turnover related to chronic inflammation, increased intestinal permeability to oncogenic factors, malabsorption of protective factors such as vitamins A and E, and impaired immunogenic surveillance in CD are considered in the pathogenesis of this complication [31]. SBA is usually diagnosed at an advanced stage (74% in stage III or IV) [29] because of late-presenting symptoms. Three of the four SBA cases were at stage III, one at stage II, and none at stage IV. Abdominal pain, gastrointestinal bleeding, vomiting, signs of ileus and bowel perforation, weight loss, and anemia are the most frequent symptoms of the disease. In our four patients with SBA, three had abdominal pain, two presented with anemia, and one presented with vomiting. The tumor is most frequently detected in the duodenum (55%) and jejunum (30%) and less frequently in the ileum (15%) [32]. However, adenocarcinoma complicating CD is most frequently detected in the jejunum [33] and is unreachable by standard upper endoscopy. In four patients with adenocarcinoma, two had infiltration of the duodenum, and two had infiltration of the jejunum. Enteroscopy, MR, or CT enterography are used in the diagnostic process. Video capsule enteroscopy is discouraged by some authors due to the risk of capsule retention and the inability to collect mucosal samples [34].

The therapy for SBA follows the guidelines for CRCA therapy. Surgery is the only potentially curative method; however, 39% of patients relapse after resection. Adjuvant chemotherapy based on fluoropyrimidine can be discussed in stage III and in stage II for T4 [35]. The programmed cell death protein-1 (PD-1)/programmed cell death/ligand 1 (PD-L1) pathway is a possible therapeutic target, with the possibility of using checkpoint inhibitors in CD-associated SBA [36]. Prognosis is generally poor, with median overall survival of 20.1 months and 5-year overall survival of 26% [37]. However, survival is better when assessing CD-associated SBA, with 5-year overall survival of 64.2% and 83% in two studies [38, 39]. One patient in our cohort died 9 months after diagnosis without therapy. Of the three patients who underwent resection and adjuvant chemotherapy (combined with stereotactic radiotherapy in one case), two were in remission for 36 and 50 months, while one relapsed after 12 months and died 22 months after diagnosis.

Information on the incidence of oropharyngeal carcinoma in CD is sparse. One study reported an increased risk of oropharyngeal cancer in patients hospitalized for CD with SIR of 2.3 [7]. Another study mentioned an increased risk of mouth and pharyngeal cancer with RR 9.7, falling to the risk of the general population after 5 years of GFD [21]. Our patient had diagnoses of CD and gastroesophageal reflux disease, a risk factor for oropharyngeal cancer [40, 41].

Most studies comparing the risk of CRCA in CD concluded that the cancerous risk is not increased [8, 42–44]. Only one study showed a marginally increased risk with SIR 1.5 [7]. Our patient was 46 years of age at the time of CRCA diagnosis. She had no risk factors for CRCA nor a family history.

The association of B-cell lymphoma with CD is not as strong as that of EATL. In a study assessing the development of B-cell lymphoma in CD, the SIR was 2.2 [3]. However, as the incidence of B-cell lymphoma is much higher (incidence of DLBCL, the most frequent B-cell lymphoma, is 7/100,000 persons/year [45]) than that of EATL, it occurs more frequently in patients with CD and is more clinically relevant than EATL. DLBCL is the most frequent type of B-cell lymphoma seen in patients with CD [2–4]. B-cell lymphoma can occur in the gut but is more frequently of non-gastrointestinal origin [3]; thus, the symptoms vary. Our patient had DLBCL localized in the duodenum. In one study, most cases (75%) had limited disease (stage I or II). The median age at diagnosis of B-cell lymphoma is 65 years [3]; however, some studies have described B-cell lymphoma in patients aged 30–39 years [2]. Our patient was diagnosed at 73 years with stage IVB. Therapy of B-cell lymphoma is based on chemotherapy and, less frequently, radiotherapy or a combination. DLBCL is usually treated using the CHOP or R-CHOP regimen. Our patient received solumedrol, rituximab, and 6 cycles of R-CHOP. The prognosis of B-cell lymphoma complicating CD is more favorable than that of EATL. In one study, two-thirds of patients responded to first-line therapy with relapse-free survival of 26 months [4]. In another study, the mean survival time was 6 years, and 5-year survival was 44% [3]. Our patient has been in remission for 45 months.

EATL required mentioning to fulfill this review, even if it was not detected in our patients. EATL is a rare lymphoma that accounts for less than 1% of non-Hodgkin lymphomas [46]. The estimated incidence of EATL is 1–1.4/1,000,000 persons/year, and it is most frequently diagnosed in the sixth decade of life [18]. There are two types of EATL. Type I (80–90% of all cases) is associated with CD with a SIR as high as 24 and an odds ratio of 28 [1, 47, 48], and often progresses from refractory CD type II [49, 50]. The possible explanation for this association is chronic inflammation, which is supported by a higher incidence of persistent villous atrophy [34]. Type II EATL arises *de novo* [18, 47]. Most EATL cases (70–90%) are diagnosed in the late stage of the disease [44].

EATL is most frequently found in the small intestine, specifically the jejunum, then the ileum and duodenum [51]. However, due to extraintestinal dissemination of aberrant intraepithelial lymphocytes, it could also be found in the large intestine, stomach, lymph nodes, bone marrow, skin, and lungs [52]. Diarrhea, abdominal pain, weight loss, vomiting, fever, night sweats, anemia, and peritonitis are the most frequent symptoms in the case of intestinal localization. Enteroscopy, MR enterography, and PET/CT are used to diagnose EATL and screen patients with refractory CD type II. These methods could also help to localize tumors for biopsy. Biopsy could be navigated by CT or performed during device-assisted enteroscopy or explorative laparotomy. Lymphoma has macroscopic features in the form of ulcerations, nodules, or large tumor masses, and microscopic features such as medium-to-large-sized cells with medium-sized, round, darkly staining nuclei with a rim of pale cytoplasm. Malignant cells are CD3, CD7, and CD103 positive and CD5 negative, variable for CD8 and TCRβ, and more than 80% of cells are CD30 positive [18, 53]. Chemotherapy based on anthracycline, especially the CHOP regime, is considered standard therapy. Resection of huge exulcerated tumors of the small intestine is recommended to reduce the risk of perforation and bleeding during chemotherapy [18]. Nevertheless, the prognosis is very poor, with a median overall survival of 7.1–10 months [18]. High-dose chemotherapy combined with autologous hematopoietic stem cell transplantation is the preferred option in select patients [49]. Responsiveness to this therapy is described in 30–60% of cases [49, 53]. Brentuximab, a chimeric anti-CD30 antibody, may be considered in patients unable to undergo or tolerate standard chemotherapy [26].

To summarize, we have reported seven cases of malignancies in patients with CD. Four patients were diagnosed with SBA, the most frequent complication in our cohort. Additionally, there was one case of DLBCL. It was unclear whether these tumors were connected to CD in two cases (carcinoma of the tongue and CRCA). Nevertheless, malignancies occurred in 7 of 190 patients, which accounts for 3.7%. Awareness of malignant complications of CD, risk factors, presentation, and disease course could lead to earlier diagnosis and improved outcomes.

## Data Availability

All data generated or analysed during this study are included in this published article and its additional information files.

## Abbreviations

CD: Celiac disease
a-TTG: Anti-tissue transglutaminase
a-DGP: Anti-deaminated gliadin peptides
GFD: Gluten-free diet
RR: Relative risk
EATL: Enteropathy associated T-cell lymphoma
CT: Computer tomography
PET/CT: Positron emission tomography/computer tomography
XELOX: Capecitabine plus oxaliplatin
DLBCL: Diffuse large B-cell lymphoma
R-CHOP: Rituximab–cyclophosphamide–doxorubicine–vincristine–prednisolone
MR: Magnetic resonance
SBA: Small bowel adenocarcinoma
PD-1: Programmed cell death protein-1
PD-L1: Programmed cell death-ligand 1
